# Xenogenic Implantation of Equine Synovial Fluid-Derived Mesenchymal Stem Cells Leads to Articular Cartilage Regeneration

**DOI:** 10.1155/2018/1073705

**Published:** 2018-06-06

**Authors:** Mohammed Zayed, Steven Newby, Nabil Misk, Robert Donnell, Madhu Dhar

**Affiliations:** ^1^Department of Large Animal Clinical Sciences, College of Veterinary Medicine, University of Tennessee, Knoxville, TN 37996, USA; ^2^Comparative and Experimental Medicine, College of Veterinary Medicine, University of Tennessee, Knoxville, TN 37996, USA; ^3^Department of Surgery, College of Veterinary Medicine, Assuit University, Asyut 71526, Egypt; ^4^Biomedical and Diagnostic Services, College of Veterinary Medicine, University of Tennessee, Knoxville, TN 37996, USA

## Abstract

Horses are widely used as large animal preclinical models for cartilage repair studies, and hence, there is an interest in using equine synovial fluid-derived mesenchymal stem cells (SFMSCs) in research and clinical applications. Since, we have previously reported that similar to bone marrow-derived MSCs (BMMSCs), SFMSCs may also exhibit donor-to-donor variations in their stem cell properties; the current study was carried out as a proof-of-concept study, to compare the in vivo potential of equine BMMSCs and SFMSCs in articular cartilage repair. MSCs from these two sources were isolated from the same equine donor. In vitro analyses confirmed a significant increase in COMP expression in SFMSCs at day 14. The cells were then encapsulated in neutral agarose scaffold constructs and were implanted into two mm diameter full-thickness articular cartilage defect in trochlear grooves of the rat femur. MSCs were fluorescently labeled, and one week after treatment, the knee joints were evaluated for the presence of MSCs to the injured site and at 12 weeks were evaluated macroscopically, histologically, and then by immunofluorescence for healing of the defect. The macroscopic and histological evaluations showed better healing of the articular cartilage in the MSCs' treated knee than in the control. Interestingly, SFMSC-treated knees showed a significantly higher Col II expression, suggesting the presence of hyaline cartilage in the healed defect. Data suggests that equine SFMSCs may be a viable option for treating osteochondral defects; however, their stem cell properties require prior testing before application.

## 1. Introduction

The regeneration capability of articular cartilage is limited due to the lack of blood vessels and nerve supply [1]. Bone marrow stimulation techniques such as subchondral drilling, abrasion, and microfracture procedures are the currently accepted methods of regenerating articular cartilage defects which aim at employing bone marrow constituents to repair the defects [2, 3]. These procedures are supposed to stimulate chondrogenesis coupled to the formation of fibrocartilage and/or hyaline cartilage. Autologous chondrocyte implantation is another technique which has been used in the repair of chondral and osteochondral lesions [4, 5]. This technique however, suffers from technical and biological challenges, including site morbidity, low numbers of chondrocytes, and the formation of the undesirable fibrocartilage [6, 7]. Regeneration of articular cartilage is thus a health concern for both human and veterinary patients, and an ideal therapy has yet to be identified.

Recent literature suggests that mesenchymal stem cells (MSCs) may present an attractive treatment option for articular cartilage repair [8]. Mesenchymal stem cells can be easily isolated from different adult tissue sources, such as bone marrow, adipose tissue, umbilical cord blood, or synovium [9] and can differentiate into chondrocytes under optimal conditions when stimulated by growth factors [10, 11]. Bone marrow-derived MSCs (BMMSCs) have acquired special attention for cartilage regeneration; it has proven to promote cellular proliferation while preserving the chondrocyte phenotype [12, 13]. However, recent reports have suggested that synovial fluid-derived MSCs (SFMSCs) may have the potential and hence may be a better source to regenerate cartilage defects, such as those involving chondral and osteochondral defects [14]. Synovial fluid-derived MSCs present an attractive cell source because they can be harvested relatively in a minimally invasive manner from synovial fluid and retain a particularly high capacity for chondrogenic differentiation and proliferation compared with MSCs obtained from other tissues, such as bone marrow or periosteum [9, 15, 16].

Horse joints are anatomically equivalent to the human knee and ankle, and as a result, horses are widely used as large animal preclinical models for cartilage repair studies. Specifically, the equine chondral defect models have been recognized to have specific advantages for translation into human articular cartilage regeneration [17–19]. While horses may more closely approximate the human clinical situation, large animal studies pose logistical and financial challenges. As a result, small animal rodent models are cost effective and have proven to be useful for proof-of-concept studies. Additionally, rodent models allow for the use of xenogenic cells and hence present methods that can be used for further experimentation in large animal models.

We previously reported a donor-matched comparison of bone marrow and synovial fluid-derived MSCs from 5 healthy mixed breed horses (age 8–13 years) with respect to their *in vitro* properties of proliferation, expression of mesenchymal stem cell protein markers, and chondrogenic potential. Out of the 5 donors, we identified one in which the SFMSCs were similar to the BMMSCs in their proliferation and expression of CD29, CD44, and CD90 but exhibited a significantly higher *in vitro* chondrogenesis [16]. Based on the *in vitro* results, we hypothesized that the SFMSCs will have a significantly higher chondrogenic potential *in vivo* as well. To prove our hypothesis, in this study, we compared the *in vivo* chondrogenic potential of SFMSCs and BMMSCs by implanting them in a rat osteochondral defect model and evaluated articular cartilage healing.

## 2. Materials and Methods

### 2.1. Cell Culture, Protein Extraction, and Western Blot (WB) Analysis of Equine MSCs

BMMSC and SFMSC cultures have been previously generated and characterized in our laboratory [16, 20]. Cryopreserved passage 3 MSCs from one specific equine donor were used in all the experiments in this study.

For chondrogenic differentiation, BMMSCs and SFMSCs were cultured in DMEM F12 media supplemented with transforming growth factor beta 1 (TGF-*β*1) (R&D Systems, Minnesota, USA) for 14 days. For WB analyses, total cell lysates were prepared using the standard radioimmunoprecipitation (RIPA) buffer composed of 50 mM Tris-HCl (pH 7.5), 1% Nonidet P-40, 0.25% Na deoxycholate, 150 mM NaCl, 1 mM EDTA (Boston BioProducts, Ashland, Massachusetts, USA), and complete protease inhibitor cocktail. Cells were harvested, and after sonication, the supernatant was collected by centrifugation and the protein concentration was determined using the bicinchoninic acid (BCA) protein assay (Thermo Scientific, Rockford, Illinois, USA). For WB analysis, 50 *μ*g of each protein sample was electrophoresed on a 10% polyacrylamide gel and the separated proteins were transferred onto a nitrocellulose membrane, subsequent to which specific proteins were identified. For each analysis, 3 *μ*g of anticartilage oligomeric matrix protein/thrombospondin-5 (COMP/TSP5) (Thermo Fisher Scientific, Rockford, Illinois, USA) was used to detect the target protein. Antigen detection was performed using secondary horseradish peroxidase-conjugated antibody (Cell Signaling Technology, Danvers, Massachusetts, USA) followed by exposure to ECL-2 reagent (Pierce, Thermo Scientific, Rockford, Illinois, USA). The intensity of the signal obtained for each protein was normalized to that of *β*-tubulin (Santa Cruz Inc., Dallas, Texas, USA) and was quantified by densitometry using ImageJ software (version 1.48, imagej.nih.gov).

### 2.2. MSC and Scaffold Constructs

Cryopreserved passage 3 cells were thawed quickly in a 37°C water bath and washed with HBSS. MSCs were labeled for cell tracking by the fluorescent lipophilic tracer 1,1′-dioctadecyl-3,3,3′,3′-tetramethylindocarbocyanine perchlorate (DiI; Molecular Probes). For labeling, 1 × 10^6^ cells/mL were incubated with 50 *μ*g/mL CM-DiI for 15 min at 37°C. Excess label was removed by washing with HBSS. Labeled cells were then mixed with an equal volume of sterile neutral 2% (*w*/*v*) low-melting point agarose (Affymetrix Inc.) solution. On gelling, agarose and MSC scaffolds were formed which could be used for implantation.

Cellular proliferation and viability of cultured MSCs within the agarose scaffold were evaluated prior to *in vivo* implantation. The proliferation rate of expanded MSCs was evaluated at 3 and 14 days after seeding, using the CellTiter 96 Aqueous Non-Radioactive (MTS) assay (Promega, Madison, Wisconsin, USA), and data was obtained as described earlier [16, 21].

Cell adhesion and viability were also evaluated microscopically after 3 and 14 days by means of calcein-AM (Invitrogen, Eugene, OR, USA) and propidium iodide (Invitrogen, Carlsbad, CA, USA) staining. MSCs were seeded at a density of 2.0 × 10^4^ cells per well per agarose plug in a 24-well plate. Cells were stained as per the manufacturer's protocols and consequently visualized using a Zeiss Axiovert 40 C microscope (Carl Zeiss MicroImaging Inc., Thornwood, New York, USA) equipped with a Nikon Digital Sight DS-Qi1Mc camera (Nikon Instruments Inc., Melville, New York).

### 2.3. Animals

All experiments were conducted in accordance with the institutional approved protocol. Twelve-week-old adult Sprague-Dawley rats (0.22–0.25 kg) were used in all experiments. Rats were acclimated in wire cages in hygienic ventilated animal rooms with controlled temperature, humidity, and 12-hour light/dark cycles for one week before use. Rodent chow and water were provided ad libitum.

### 2.4. Surgery

Anesthesia was induced by inhalation of a 2% isoflurane/oxygen gas mixture. Before surgery, each animal received a subcutaneous injection of buprenorphine (0.05 mg/kg) and eye lubricant. Enrofloxacin was provided in drinking water for each animal for at least 3 days postoperatively.

**Figure 1:**
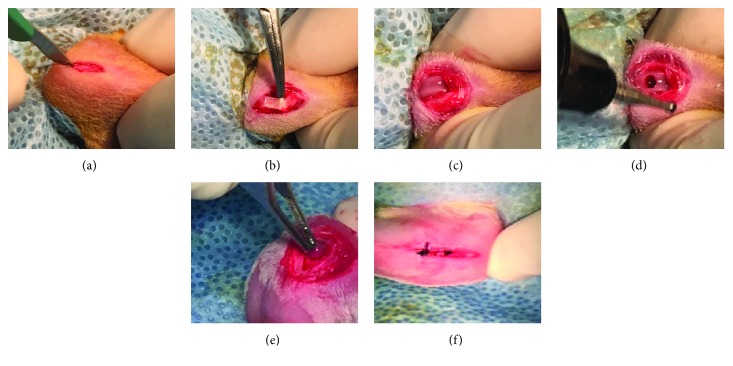
Surgical procedure showing the creation of the osteochondral defect. (a) Both knee joints were opened using an anteromedial approach. (b), (c) The patellae and tendon were laterally dislocated to expose the articular cartilage. (d) 2 mm diameter full-thickness articular cartilage defects were created in trochlear grooves of the distal femur by carefully drilling in a vertical direction using a power microdrill. (e) The BMMSCs or SFMSCs (3.0 × 10^6^ cells) and agarose scaffolds were implanted into the defects in the experimental (left, treated) knee. The scaffold only (agarose) was implanted in the control right knee. (f) The arthrotomy was closed with interrupted 4–0 nylon sutures, and the skin was closed with continuous 4–0 nylon sutures.

For surgery, after the sterile preparations of the surgical site, both knee joints of each rat were opened through an anteromedial approach. The patellae and tendon were laterally displaced, and 2 mm diameter full-thickness articular cartilage defects were created in the trochlear grooves of the distal femur using a power drill. The defect was roughly 3 mm deep through the subchondral bone (Figure 1). After removing the cartilage and bone debris, the defects were irrigated with sterile saline, and the BMMSCs or SFMSCs and agarose constructs containing 3 × 10^6^ cells each were implanted. The left knee was used as the treatment knee, and agarose alone was placed into the right knee, which served as the control. Rats were sacrificed at a one-week time point to track the labeled cells, and harvested samples at 12 weeks were used to evaluate cartilage healing.

### 2.5. Macroscopic and Histological Evaluations

Rats were sacrificed at 1 and 12 weeks postsurgery, and the joints were harvested, fixed in 10% formaldehyde, decalcified in 10% nitric acid for two days, dehydrated in graded ethanol, and finally embedded in paraffin wax. Paraffin-embedded samples were then cut into 5 *μ*m sections for evaluation.

The 1-week samples were visualized to track the DiI-labeled MSCs and were stained with the histological stain hematoxylin and eosin (H&E). DiI fluorescence was visualized under UV, and images were captured using NIS-Elements 3.10 (Nikon).

The 12-week samples were analyzed using H&E and Masson's trichrome stains (Sigma, St. Louis, MO, USA) and by immunofluorescence to evaluate the expression of collagen type II (Abcam, USA). H&E staining was carried out as previously described [22]. For Masson's trichrome staining, tissue sections were stained in Masson's composition solution for five minutes and differentiated in 5% phosphotungstic acid for ten minutes. Tissue sections were then stained in aniline blue solution for 5 min, and extra stain was removed by rinsing with 0.2% acetic acid. The specimens were graded semiquantitatively by a trained pathologist, who was blinded to the identity of each sample. The scoring scale was based on the filling of the defect, cell morphology, and inflammatory response, as described [23].

Type II collagen was detected by immunofluorescence. Sections were deparaffinized, washed with HBSS, permeabilized with 0.1% Triton X-100 (Sigma) for 10 min, and blocked with 1% Power Block (BioGenex) for 30 min at room temperature. Sections were incubated with anti-type II collagen monoclonal antibody (1 : 200; Abcam, USA) at 4°C overnight. After washing with HBSS, the sections were incubated for 30 min with Alexa Fluor 488 anti-rabbit secondary antibody. Samples were mounted in ProLong Gold antifade reagent with DAPI (Life Technologies), and images were obtained with a laser scanning spectral confocal microscope (Leica TCS SP8; Leica Microsystems©, Wetzlar, Germany). Mean intensity fluorescence was measured using the Leica TCS SP2 software by selecting at least four representative fields of identical settings.

### 2.6. Statistical Analysis

All quantitative group data are shown as the mean ± standard deviation (SD). Immunoblot and immunofluorescence data were analyzed by repeated measures one-way analysis of variance (ANOVA). SAS 9.4 (SAS Inc., NC, USA) statistical software package was used. Differences of *P* < 0.05 were considered to be statistically significant.

## 3. Results

We have previously reported a donor-matched comparison of bone marrow and synovial fluid-derived MSCs from 5 healthy mixed breed horses (age 8–13 years) with respect to their *in vitro* properties of proliferation, their expression of protein markers, and finally their chondrogenic potential. Using these assays, we identified a donor, whose SFMSCs were similar to the BMMSCs in their proliferation and expression of CD29, CD44, and CD90 but exhibited a significantly higher chondrogenic potential based on the increased expression of aggrecan and type II collagen proteins [16]. The next step is to evaluate the chondrogenic potential of the SFMSCs and BMMSCs in an *in vivo* model, prior to their application in equine clinical cases of articular cartilage injuries.

In the current study, we report the *in vivo* evaluation of the SFMSCs and BMMSCs from a single donor described above for their potential to regenerate articular cartilage in an osteochondral defect in a rat model. In this study, the rat model serves as an animal model to evaluate the *in vivo* chondrogenic potential of MSCs from two tissue sources. This project was initiated with a hypothesis that the SFMSCs which have a higher chondrogenic potential *in vitro* will exhibit a higher potential to heal damaged articular cartilage *in vivo*. Primary cultures of BMMSCs and SFMSCs generated from a specific donor described earlier [16] were used.

### 3.1. *In Vitro* Chondrogenesis

**Figure 2:**
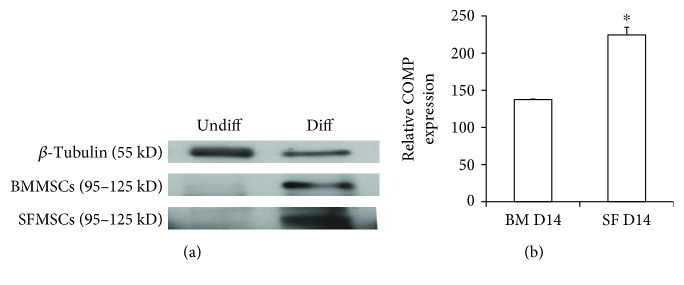
Immunoblot analyses of COMP proteins. (a) Western blot analysis showed expression of COMP in *in vitro* differentiated BMMSCs and SFMSCs. (b) COMP expression was significantly higher in differentiated SFMSCs suggesting a relatively higher chondrogenic potential. The asterisk indicates (*P* < 0.05). *β*-Tubulin was used as a control, and the expression of *β*-tubulin was used to normalize the expression of COMP in each sample. Each experiment was carried out with two independent protein samples, each sample analyzed in duplicate.

Previously isolated, characterized, and cryobanked passage 3 SFMSCs and BMMSCs were first evaluated by *in vitro* chondrogenic differentiation. The expression of cartilage oligomeric matrix protein (COMP) was used as an indicator of the chondrogenic differentiation process. MSCs from both cell sources displayed efficient chondrogenesis in a period of 14 days as per the conditions reported earlier [16]. Qualitative and quantitative immunoblot analyses showed that COMP expression increased with increase in cell differentiation from both sources. As expected from the previously published report, SFMSCs exhibited a significant increase compared to BMMSCs at day 14 when the chondrogenic differentiation was complete. Results indicate and confirm that there was a significant increase in the proteoglycan deposition when SFMSCs differentiated *in vitro* as compared to BMMSCs obtained from the same donor (Figure 2).

### 3.2. Agarose Scaffold Conserves Proliferation and Viability of MSCs

**Figure 3:**
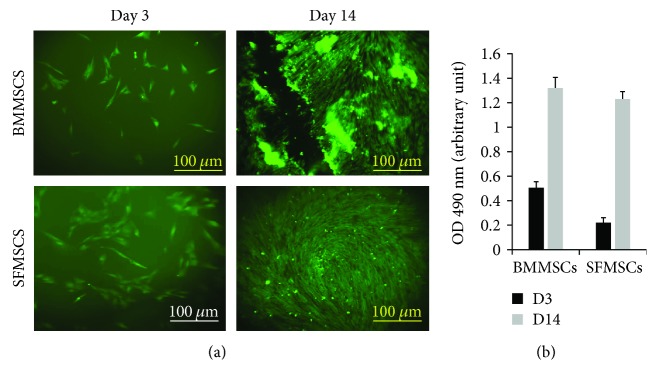
Proliferation and viability of MSCs. (a) Representative fluorescence microscope images of MSCs cultured for 3 and 14 days on agarose with live (green)/dead (red) stain. (b) Proliferation rate of MSCs cultured for 3 and 14 days on agarose. Absorbance, that is, optical density at 490 nm, is linearly related to the cell numbers, and hence, the value represents cell numbers at a given time point, and comparison of these values at two different time points is used as an indicator of proliferation. Data is presented as means ± SD. Error bars represent the SD.

Once we confirmed the chondrogenic potentials of MSCs, an agarose scaffold was designed to serve as a vehicle to deliver MSCs *in vivo*. Prior to *in vivo* implantation, proliferation and viability of MSCs within the agarose construct were assessed through 14 days using live-dead staining and MTS assay (Figure 3). Cell viability was verified in the agarose construct using calcein AM and propidium iodide fluorescent (live-dead) staining (Figure 3(a)). Additionally, the MSCs exhibited a linear increase in proliferation between days 3 and 14, indicating a linear increase in cell number with time (Figure 3(b)). Data showed that MSCs seeded on agarose construct were metabolically active and viable in the presence of agarose, thus confirming the biocompatibility of the agarose scaffold.

### 3.3. Implanted MSCs “Home” to the Defect Site *In Vivo*

**Figure 4:**
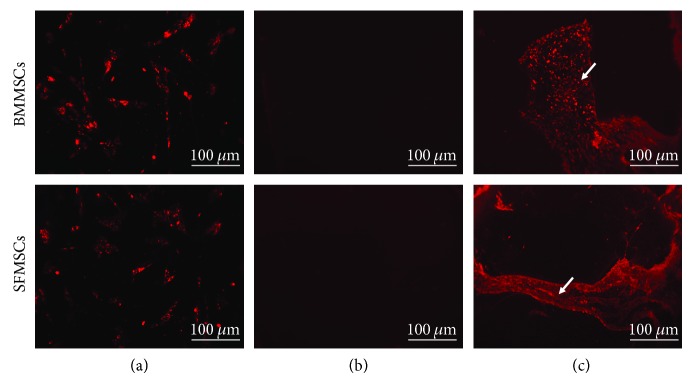
Fluorescent imaging of MSCs. Representative images to show (a) DiI-positive MSCs before implantation. (b) Absence of DiI-positive MSCs in the control group, and (c) DiI-positive MSCs in the treated groups. Arrows indicate the persistence of DiI-labeled MSCs at least 1 week postimplantation, suggesting the localization of cells at the injury site.

To demonstrate the presence and assess the time that the MSCs remain at the site of the defect, CM-DiI labeled cells were tracked *in vivo*. Fluorescence imaging showed the presence of CM-DiI-labeled cells within the defects of both the SFMSC- and BMMSC-treated rats. Cells were visualized at one week following implantation (Figure 4). None of the CM-DiI-labeled cells were detected in the harvested samples at 12 weeks. In contrast, control specimens did not show any CM-DiI fluorescence, thus confirming the presence and “homing” of MSCs at the defect site for at least 1 week postimplantation.

### 3.4. Macroscopic and Histomorphometric Analyses

**Figure 5:**
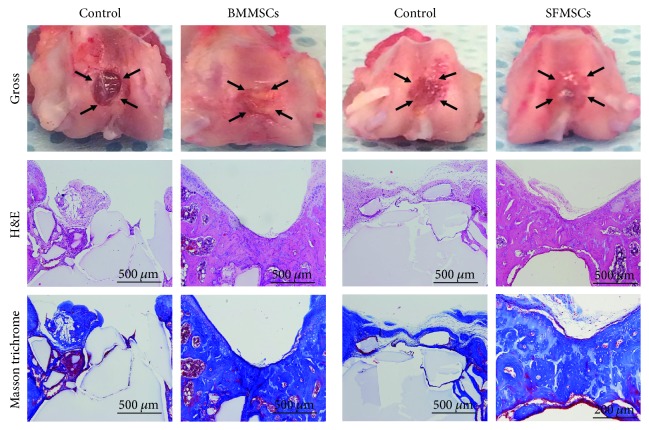
Histological and gross analyses. Gross appearance of the osteochondral defect twelve weeks after treatment with BMMSCs or SFMSCs, the arrowheads point to the edges of the defect (top panel). Representative histological results using light microscopy twelve weeks posttherapy. H&E staining (middle panel) and Masson trichrome staining (bottom panel) illustrate lack of “abnormal” cells and collagen growth in the defect.

Inflammation, rejection, and existence of any depression or bulging of repaired tissues in the defect area or any other abnormality were grossly evaluated. Gross observations did not show any inflammation or fibrosis, indicating lack of any infection due to MSCs or agarose or both throughout the study period, further confirming the biocompatibility of the scaffold. At 12 weeks after treatment, the defect surfaces were still depressed in treated knees but were filled compared to the control. Interestingly, agarose remnants were still visible in the defect even after 12 weeks, which helped identify the defects stained with H&E and Masson trichrome (Figure 5).

Using H&E-stained specimens, we confirmed that there was no inflammatory reaction in the treated knees either at 1 week or at 12 weeks. Only three rats showed mild inflammation at 12 weeks, and they were removed from further analyses. Masson trichrome-stained specimens in the control knees did not demonstrate any signs of newly formed collagen in the defect, suggesting lack of cartilage repair compared to treated knees (Figure 5).

**Figure 6:**
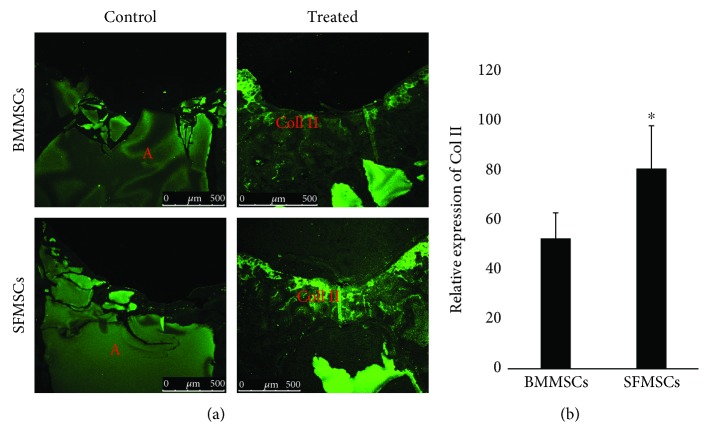
Representative images and quantitation to assess collagen II posttherapy. Immunofluorescence staining of type II collagen (Col II) at the articular cartilage defect in the rat femoral trochlear groove at 12 weeks after implantation of BMMSCs and SFMSCs (left panel). Relative expression of Col II in treated knees (right panel) suggests a statistically higher level of expression in the healed defect. Asterisk represents statistically significant increase in Col II (*P* < 0.05). A = agarose; Col II = collagen type II.

*In vivo* immunofluorescence staining for Col II in BMMSC- and SFMSC-treated samples showed strong staining distributed in the defect area compared to control knees. The MSC-treated knees in each group showed significantly higher expression of type II collagen than the corresponding controls, with the SFMSCs showing significantly higher intensity, indicating a significant increase in the production of hyaline cartilage compared to BMMSC-treated joints (Figure 6).

## 4. Discussion

In the current study, we aimed to evaluate and compare SFMSCs as a new cell source, in relationship to BMMSCs, which is considered a standard cell source for articular cartilage regeneration. While carrying out these comparisons, we wanted to confirm that the *in vitro* properties of MSCs are dependent on the donor and are indeed translated into an *in vivo* setting and that the *in vitro* properties can provide a very reliable indication of their *in vivo* action.

Articular cartilage is a highly differentiated, avascular tissue with very little self-regeneration capacity. Autologous chondrocyte implantation used to increase the repair potential of damaged cartilage showed a significantly better histological score compared to microfracture or osteochondral autologous graft [24–26]. Complications accompanying with cell expansion, graft collapse, and tissue hypertrophy still pose challenges [27]. Adult MSCs are an attractive cell source for cell-based strategies in regenerative medicine. MSCs, specifically those derived from the synovial tissues, have been demonstrated to enhance the quality and quantity of the repaired tissue in full-thickness articular cartilage defects [28, 29]. Published reports including one from our laboratory showed that SFMSCs are proliferative and possess chondrogenic potential, and hence, enough cell numbers can be obtained to treat an articular cartilage injury [16, 30, 31]. It is reported that SFMSCs increase in the knee with degenerated cartilage in OA patients [32], suggesting a physiological role of synovial MSCs during cartilage recovery.

Although both bone marrow and synovial fluid represent an attractive tissue source for MSCs and have the potential to treat cases with articular cartilage injuries, results from our laboratory have shown that the properties of MSCs are donor-dependent, which could potentially affect their clinical efficacy (Carter et al. 2013). In this study, we confirmed that as observed earlier, there was a significant increase in COMP expression of SFMSCs than BMMSCs, indicating more matrix formation and hence a higher chondrogenic potential [9, 33]. We were able to demonstrate increased chondrogenesis of SFMSCs by evaluating the expression patterns of two chondrocyte marker proteins, COMP and Col II. COMP is an abundant cartilage extracellular matrix protein that interacts with major cartilage components including aggrecan and collagens. The COMP expression increases during chondrogenic differentiation and is a good indicator of cells undergoing chondrogenesis [34]. Type II collagen or Col II is the principal component of the extracellular matrix of adult articular cartilage and hence is a good marker to evaluate articular cartilage repair. Most importantly, the presence of Col II in regenerated cartilage indicates the presence of hyaline cartilage, thus suggesting healthy tissue.

Biomaterials play an important role as a delivery vehicle in cell transplantation as well as in providing an initial three-dimensional structure for complex tissues with essential geometry. A biomaterial which is biocompatible, exhibits deposition of extracellular matrix and which promotes chondrogenic protein expression, is a good candidate for engineering cartilaginouse tissues. Agarose is a saccharide polymer isolated from sea algae, which is gradually desorbed mostly by macrophage phagocytosis and enzymatic damage [35]. Agarose is one of these biomaterials that have been suggested to serve as a cell carrier of MSCs for cell transplantation and has been shown to sustain and support chondrocyte viability and phenotype *in vitro* and *in vivo* and is also inert, and hence, will not be bioactive [36–39].

In our study, MSCs could maintain good proliferation and viability in the presence of 2% neutral agarose. These data were in agreement with the reports presented by others and our laboratory [22, 36].

The xenogenic rat model for this study has been described previously [40]. As expected, even though we used xenogeneic MSCs in this model, we did not observe any immune response to the equine cells in the treated knees. In fact, we detected hyaline-like cartilage formation in these samples, demonstrating that the implanted cells were favorable and had the potential for tissue repair. Our results are supported by other published studies, which have reported comparable success with xenogeneic MSCs in tissue defects without eliciting any immune response [41–43].

In this study, we quantified the cartilage regeneration based on the filling of the defect area, matrix, and hyaline cartilage formation. As hypothesized, SFMSCs led to enhanced cartilage repair *in vivo*, and the regenerated area in the MSC-treated knees was significantly higher than that in the control knees after 12 weeks. Most importantly, there was a significant increase in Col II expression in SFMSCs compared to BMMSCs at 12 weeks. In summary, these results demonstrated that implantation of synovial MSCs promoted cartilage regeneration [31, 44].

Cell adhesion to the extracellular matrix is an essential activity in tissue organization. One week after MSC implantation, cartilage defects were already supplied with MSCs, whereas the DiI-positive area was detected histologically. In the radial histological section, DiI-labeled cells were located mostly in the center of the defect. We examined the location of DiI-labeled cells at 12 weeks, and thereafter, we could not find any. These findings indicate that implanted MSCs adhered around the defect of cartilage at the first stage; after that, the newly formed chondrocytes differentiated into cartilage tissue [45]. It is still unknown whether the MSCs trigger the healing by their paracrine effect on the progenitor cells of the injured area or they themselves undergo differentiation. Using this model and the system described in our study, we can now investigate these mechanisms.

## 5. Conclusion

In this study, we demonstrate that implantation of SFMSCs was successful in terms of *in vivo* evaluation and Col II expression. The use of SFMSCs is advantageous where the cells can be ready at passage 0 in the first two weeks postharvest and hence can be applied in an autologous or allogeneic manner within a short time period. Implantation of SFMSCs can be less aggressive compared to the other techniques. In this study, we used xenogeneic cells; hence, future experiments involving the implantation of equine MSCs into an articular cartilage defect in a horse model should be carried out to confirm the results and thus investigate the effectiveness of this treatment.

## Data Availability

The data used to support the findings of this study are available from the corresponding author upon request.

## Abbreviations

BMMSCs: Bone marrow mesenchymal stem cells
Col II: Collagen type II
COMP: Anticartilage oligomeric matrix protein
GAG: Glycosaminoglycan
IF: Immunofluorescence
MSCs: Mesenchymal stem cells
OA: Osteoarthritis
SFMSCs: Synovial fluid mesenchymal stem cells.
